# Sensitive Cr^3+^ sensor based on novel poly(luminol-*co*-1,8-diaminonaphthalene)/CeO_2_/MWCNTs nanocomposites

**DOI:** 10.1039/d4ra00542b

**Published:** 2024-02-14

**Authors:** Salsabeel Al-Sodies, Abdullah M. Asiri, M. M. Alam, Khalid A. Alamry, Mahmoud A. Hussein, Mohammed M. Rahman

**Affiliations:** a Chemistry Department, Faculty of Science, King Abdulaziz University P. O. Box 80203 Jeddah 21589 Saudi Arabia mahussein74@yahoo.com maabdo@kau.edu.sa mmrahman@kau.edu.sa mmrahmanh@gmail.com; b Department of Chemistry, Faculty of Science, Taibah University Al-Madinah Al-Munawarah 30002 Saudi Arabia; c Center of Excellence for Advanced Materials Research (CEAMR), King Abdulaziz University Jeddah 21589 Saudi Arabia; d Department of Chemical Engineering, Z. H. Sikder University of Science and Technology (ZHSUST) Shariatpur 8024 Bangladesh; e Chemistry Department, Faculty of Science, Assiut University Assiut 71516 Egypt

## Abstract

In this study, poly(luminol-*co*-1,8-diaminonaphthalene) (PLim-DAN) was synthesized and subsequently modified with MWCNTs and CeO_2_ NPs. The synthesized nanocomposites were analyzed using IR, SEM, TEM, and XRD. Furthermore, a comprehensive set of thermal behavior measurements were taken using TGA/DTG analysis. Next, the electroactivity of the developed nanocomposites was tested as an electrochemical sensor to measure the concentration of Cr^3+^ ions in phosphate buffers. The GCE adapted with the PLim-DAN/CeO_2_/CNTs-10% nanocomposite (NC) exhibited the highest current response among the other compositions and copolymers. The fabricated nanocomposite sensor showed high sensitivity, with a value of 19.78 μA μM^−1^ cm^−2^, and a low detection limit of 4.80 ± 0.24 pM. The analytical performance was evaluated by plotting a current calibration curve *versus* the concentration of Cr^3+^ ions. It was found to be linear (*R*^2^ = 0.9908) over the range of 0.1 nM to 0.1 mM, identified as the linear dynamic range (LDR). This electrochemical sensor demonstrated that it could be a useful tool for environmental monitoring by accurately detecting and measuring carcinogenic Cr^3+^ ions in real-world samples.

## Abstract 2

In this study, PLim-DAN was synthesized and subsequently modified with MWCNTs and CeO_2_ NPs. The electroactivity of the developed nanocomposites was tested as an electrochemical sensor to measure the concentration of Cr^3+^ ions in phosphate buffers.

## Graphical abstract

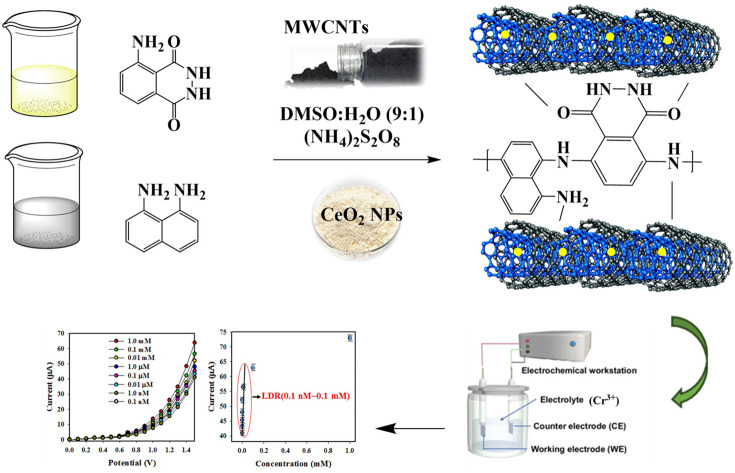

## Introduction

Considering the widespread use of polymers in various applications, scientists have focused their attention on the polymer activity of organic compounds in multiple fields. Given the need for a comprehensive understanding of these polymers, luminol polymeric moiety systems have not been studied extensively.^1^

Luminol and its derivatives have been employed in various analytical chemistry and biotechnology applications as efficient electrochemiluminescence conducting polymers.^2,3^ The primary application of electrochemiluminescence in crime scene investigations has resulted in the creation of discoveries of polymeric luminol, which enhances the efficiency, sensitivity, and quantum yield of the process.^4^ An amino group in luminol enhances chemiluminescence intensity by acting as an electron-donating group in the system. This positively impacts the electrochemical properties of poly(luminol) and its potential use in electrochemical and biosensor materials.^5–10^

From the same family, a new type of multifunctional polymer material, poly(di-aminonaphthalene), can be synthesized from aromatic diamines, such as 1,5-, 1,8-, or 2,3-diaminonaphthalene, through electrochemical or chemical oxidative polymerization with properties similar to those of polyaniline and polypyrrole.^11^ The substance 1,8-diaminonaphthalene displays properties such as electroconductivity, electrocatalysis, electroactivity, permselectivity, electrochromism, and other properties that stem from the chemical reactivity of the functional amino groups on its macromolecular structure.^12–16^

Furthermore, one of the most noteworthy and rapidly advancing fields in materials science and analytical chemistry is the design of novel electrochemical sensing materials that display exceptional electrocatalytic properties, enduring stability, consistent repetition, high reliability, and enhanced sensitivity and selectivity.^17–20^ Carbon nanotubes (CNTs) are highly valued for developing sensors and biosensors because of their remarkable chemical resistance, large surface area, high tensile strength, excellent electrical conductivity, and distinct one-dimensional structure facilitating rapid electron transfer.^21^ CNTs possess a broad spectrum of potential applications, including electronics, polymer composites, energy storage, catalysis, gas storage, and sensors.^22^ Multi-walled carbon nanotubes (MWCNTs) exhibit good mechanical strength and enhanced surface activity with a high specific surface area, making them ideal for thermally stable materials, biological applications, water filtration, structural materials, and sensors.^23,24^ Carbon nanotubes (SWCNTs and MWCNTs) have been extensively used as sensing materials to fabricate various nanocomposites that have been successfully employed to detect a wide range of bioanalytes, including uric acid, ascorbic acid, dopamine,^25^ styrene, epinephrine,^26^ glutathione, glucose,^27,28^ and toxic metal analytes, such as Ga^3+^, Fe^3+^, Hg^2+^, Pb^2+^, and Cu^2+^.^29–31^

To further improve the sensing performance, nanomaterials have been fabricated and loaded on the surface of designed polymers. Cerium oxide (CeO_2_) has garnered attention among the various nanoparticles because of its impressive properties, including proficient photocatalytic activity, high surface area, oxygen ion conductivity, high chemical stability, high specific capacitance, and non-toxicity. CeO_2_ is a rare earth metal oxide with a broadband gap (3.4 eV) and a cubic fluorite structure. Each Ce^4+^ ion was surrounded by eight O^2−^ ions in a (fcc) arrangement. Each O^2−^ ion was tetrahedrally surrounded by four Ce^4+^ ions. Consequently, CeO_2_ has been used in various applications, including solid state supercapacitors devices, solar cells, photocatalysis, and sensors.^32–38^

Heavy metals have been targeted for sensing applications due to their widespread presence as one of the most important pollutants found in water bodies and are highly toxic to ecosystems.^29–31^ Trivalent chromium (Cr^3+^) is critical in metabolizing lipids, nucleic acids, and proteins in biochemical processes, making it an essential element in trace concentrations in humans and animals.^39,40^ However, exposure to higher concentrations of Cr^3+^ ions and a deficiency of Cr^3+^ can result in diseases associated with sugar metabolism disorders, such as diabetes, cataracts, cardiovascular disease, uremia, and blindness.^41,42^ Moreover, Cr^3+^ ions can impair the quality and quantity of edible agricultural products and animals.^43^ Therefore, Cr^3+^ is considered a significant environmental pollutant and health hazard, and there is an urgent need for an easy and reliable method for detecting Cr^3+^ ions in aqueous media to ensure a sustainable environment. To this end, several efficient and sophisticated analytical methods, such as HPLC, ICP-AES, DPP, and X-ray fluorescence, have been employed to detect trace Cr^3+^ ions. However, these techniques are expensive, time consuming, and unsuitable for frequent analysis. Thus, there is a pressing need to develop a convenient probe that can rapidly and selectively detect Cr^3+^ ions using a sensor designed explicitly for cations.^44,45^

The combination of CeO_2_ NPs and MWCNTs in sensors has been reported in several studies.^29,46–51^ N. Dogra *et al.* developed a chemiresistive sensor for detecting ammonia vapor at room temperature using CeO_2_/MWCNTs composites. The resistance of the sensor increased in the presence of ammonia vapor, and the fabricated sensor had a response time of 35 s, a relatively short recovery time of approximately 100 s, high sensitivity to ammonia, and stable and reproducible characteristics over a broad range of humidity levels.^46^ Furthermore, rice-like CeO_2_/MWCNTs nanocomposite were synthesized *via* a simple hydrothermal method and were investigated by S. Shanavas *et al.* for detecting ammonia and ethanol gases at concentrations ranging from 0 to 500 ppm by employing a fiber-optic clad modification technique. The findings suggest that CeO_2_/MWCNT displays a high sensitivity of 78 counts/ppm, rapid response time of 17 s, and recovery time of 9 s in response to ethanol gas.^47^ The use of CeO_2_/MWCNTs in the gas sensor was expanded to include stretchable sensors for the detection of NO_2_. The fabricated sensor was formed by depositing the MWCNTs/CeO_2_ composites onto silicon rubber and a jelly-based substrate to develop a flexible and biodegradable sensor. The jelly-based device exhibited faster response and recovery times (22.9/345.2 s) in compared to the stretchable device.^48^ Furthermore, the two nano-structured CeO_2_/MWCNTs were successfully explored and constructed as an electrochemical sensor for neonicotinoid insecticides (nitenpyram) with a low detection limit of 0.72 μM,^49^ acetaldehyde (LOD = 7.4 nM),^50^ dopamine (LOD = 0.03 μM),^51^ and Hg^2+^, Pb^2+^, and Cu^2+^ heavy metals with LOD of 1.98, 1.10 and 3.53 μg L^−1^, respectively.^29^

As the importance of the above individual components emerged, this study investigates a sensor probe to detect Cr^3+^ ions through electrochemical means using a novel copolymer of luminol and 1,8-diaminonaphthalene. To the best of our knowledge, this is the first reported study of constructed CeO_2_/MWCNTs as a sensor for Cr^3+^ ions, as well as the polymeric luminol and 1,8-diaminonaphthalene. The copolymers modified with MWCNTs and CeO_2_ NPs enhanced the electrochemical activity, producing nanocomposites of PLim-DAN/CeO_2_/CNTs. The performance of the fabricated Cr^3+^-ion sensor with GCE was evaluated and was extremely sensitive and selective for Cr^3+^ ions. Finally, a newly developed Cr^3+^ ion sensor was applied to environmental samples to detect Cr^3+^ ions. Developing heavy metal ion sensors can provide a unique and reliable approach to environmental applications.

## Experimental

### Chemicals and reagents

Luminol, 1,8-diaminonaphthalene, ammonium persulfate (NH_4_)_2_S_2_O_8_, and dimethyl sulfoxide 98% purity (DMSO) were purchased from Aldrich. Multi-walled carbon nanotubes (MWCNTs) and cerium oxide nanoparticles (CeO_2_ NPs: 25–50 nm) were purchased from Nano Tech Co. Ltd. Egypt. Analytically graded inorganic salts of As^3+^, Cd^2+^, Co^2+^, Cr^3+^, Ga^3+^, Hg^2+^, Pb^2+^, Sb^3+^, and Sn^2+^ were obtained from the supplier, as required for this study. All chemicals were used as received without further purification.

### Instrumentation

Fourier transform infrared (FT-IR) spectra were recorded using a PerkinElmer Spectrum 100 FT-IR device in the 4000–500 cm^−1^ range. The morphologies and elemental distributions of the polymers were examined using scanning electron microscopy (SEM, TESCAN VEGA 3, Czech Republic). Samples were mounted on aluminum microscopy stubs using carbon tape and then coated with gold (Au) for 120 s using a Quorum Techniques Ltd. sputter coater (Q150t, UK). Transmission electron microscopy (TEM) Thermo Fisher Scientific, multi-purpose, Talos F200i S/TEM was used to investigate the interactions between the polymers and nanomaterials with high-resolution imaging and analysis applications operating at 200 kV. X-ray diffraction (XRD) for the designated materials data was collected using a Phillips X-ray unit (Phillips's Generator PW-1710) diffractometer with a Cu Kα irradiation source. The 2*θ* = 5–80° range was scanned at a 1° min^−1^ rate. Thermogravimetric analysis (TGA) was performed on a Shimadzu TGA 50 instrument at a heating rate of 10 °C min^−1^ in air. A Keithley electrometer was used to construct the electrochemical cell for the electrochemical (I–V) analysis.

### Synthesis of poly(luminol-*co*-1,8-diaminonaphthalene) copolymers (PLim-DAN)

The copolymer PLim-DAN was prepared in an equal ratio (1 : 1) using the original oxidative polymerization process for poly(luminol).^52^ Equipped with an N_2_ atmosphere, a mixture of H_2_O : DMSO (1 : 9) was added to luminol (1 mmol) and 1,8-diaminonaphthalene (1 mmol) in a two-neck round flask. The reaction mixture was stirred at room temperature for 30 min to ensure monomer solubility. A solution of ammonium persulfate (NH_4_)_2_S_2_O_8_ (3 mmol) was added to initiate the polymerization process. The reaction mixture was stirred for 24 h at room temperature, and the formed precipitate was collected by filtration, washed thoroughly with water multiple times, and dried at 70 °C for 48 h.

### Preparation of nanocomposite poly(luminol-*co*-1,8-diaminonaphthalene) copolymers with CeO_2_ NPs/MWCNTs

A new series of poly(luminol-*co*-1,8-diaminonaphthalene)/CeO_2_ NPs/MWCNTs was prepared *via* an *in situ* oxidative polymerization method using a fixed percentage (5%) of CeO_2_ NPs and different loadings (1, 3, 5, and 10%) of MWCNTs concerning unmodified copolymers. For each formulation, a mixture of CeO_2_ NPs (5%) and different ratios of MWCNTs in H_2_O : DMSO (1 : 9) was sonicated for 1 h, followed by the addition of equimolar amounts of the two monomers (luminol and 1,8-diaminonaphthalene), and sonication continued for 30 min. The reaction mixture was stirred for 30 min. A solution of ammonium persulfate (NH_4_)_2_S_2_O_8_ (3 mmol) was added to initiate the polymerization process for each formulation. The workup of the formed nanocomposites was similar to that of the unmodified copolymers, and the obtained composites were labeled PLim-DAN/CeO_2_/CNTs-1%, PLim-DAN/CeO_2_/CNTs-3%, PLim-DAN/CeO_2_/CNTs-5%, and PLim-DAN/CeO_2_/CNTs-10%.

### Modification of GCE with active poly(luminol-*co*-1,8-diaminonaphthalene)/CeO_2_ NPs/MWCNTs

Hare, GCE, and PLim-DAN/CeO_2_/CNTs nanocomposites were used to modify the desired electrochemical sensor using the I–V method. A slurry of PLim-DAN/CeO_2_/CNTs NCs was prepared in ethanol as a thin and uniform layer, which was subsequently deposited onto a GCE with a surface area of 0.0316 cm^2^. After drying the slurry, a drop of Nafion (5% Nafion suspension in ethanol) was added to the modified GCE to obtain the desired stability. The GCE was then thoroughly dried in an oven at 35 °C for an adequate amount of time. An electrochemical cell was assembled using a Keithley electrometer, with PLim-DAN/CeO_2_/CNTs NCs/binder/GCE as the working electrode and a simple Pt wire as the counter electrode. A chromium(iii) ion solution was prepared and used as the target analyte. A calibration curve was plotted from the linear relationship between the current and concentration of Cr^3+^ ions, and the analytical performance of the sensor, such as the sensitivity and detection limit (DL), was estimated from the slope of the calibration curve. The linear dynamic range (LDR) was determined by considering the maximum linearity (*R*^2^) of the calibration curve. During the electrochemical investigation, the phosphate buffer solution in the detection beaker was kept constant at 10.0 mL throughout the experiment. An electrochemical sensor using a Keithley electrometer is a simple two-electrode (working and counter) system.

## Results and discussion

### Chemistry

**Fig. 1:**
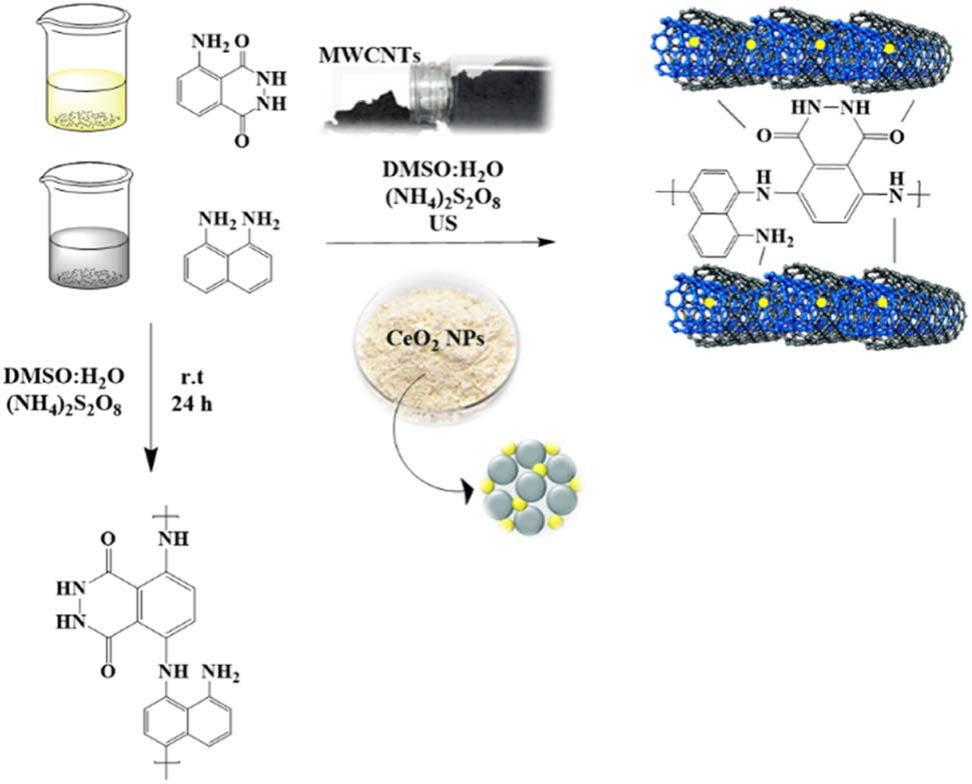
Systematic illustration of PLim-DAN and PLim-DAN nanocomposite synthesis and fabrication.

In the present work, the polymerization process consisted of two main steps: the oxidative polymerization of the genuine copolymers and the preparation of loaded nanomaterials using a typical procedure with minor modifications to distribute the nanoparticles (Fig. 1). The electrochemical performances of the designed copolymer and its composites as sensitive sensors for Cr^3+^ ions were examined. The polymerization occurred in a water and DMSO media mixture with ammonium persulfate (NH_4_)_2_S_2_O_8_ as an oxidizing agent.^12,52^ In the first approach, luminol and 1,8-diaminonaphthalene monomers were converted to polymeric form to design a novel copolymer, PLim-DAN, in an equimolar ratio under an inert atmosphere. The following process involved the modification of the pure copolymers with several ratios using a fixed percentage of cerium oxide nanoparticles (5%) (CeO_2_ NPs) and different loadings of MWCNTs (1%, 3%, 5%, and 10%). The new series of composites PLim-DAN/CeO_2_/CNTs-1%, PLim-DAN/CeO_2_/CNTs-3%, PLim-DAN/CeO_2_/CNTs-5%, and PLim-DAN/CeO_2_/CNTs-10% were produced through *in situ* oxidative polymerization by applying the same principle of pure copolymers with the introduction of the ultrasound technique to ensure complete distribution of the loaded nanoparticles.^53^ The designed copolymers were characterized using FT-IR spectroscopy, and their morphologies were studied using SEM, XRD, and TEM. At the same time, thermal behavior was investigated using TGA and derivative thermogravimetry (DTG).

### FT-IR analysis

**Fig. 2:**
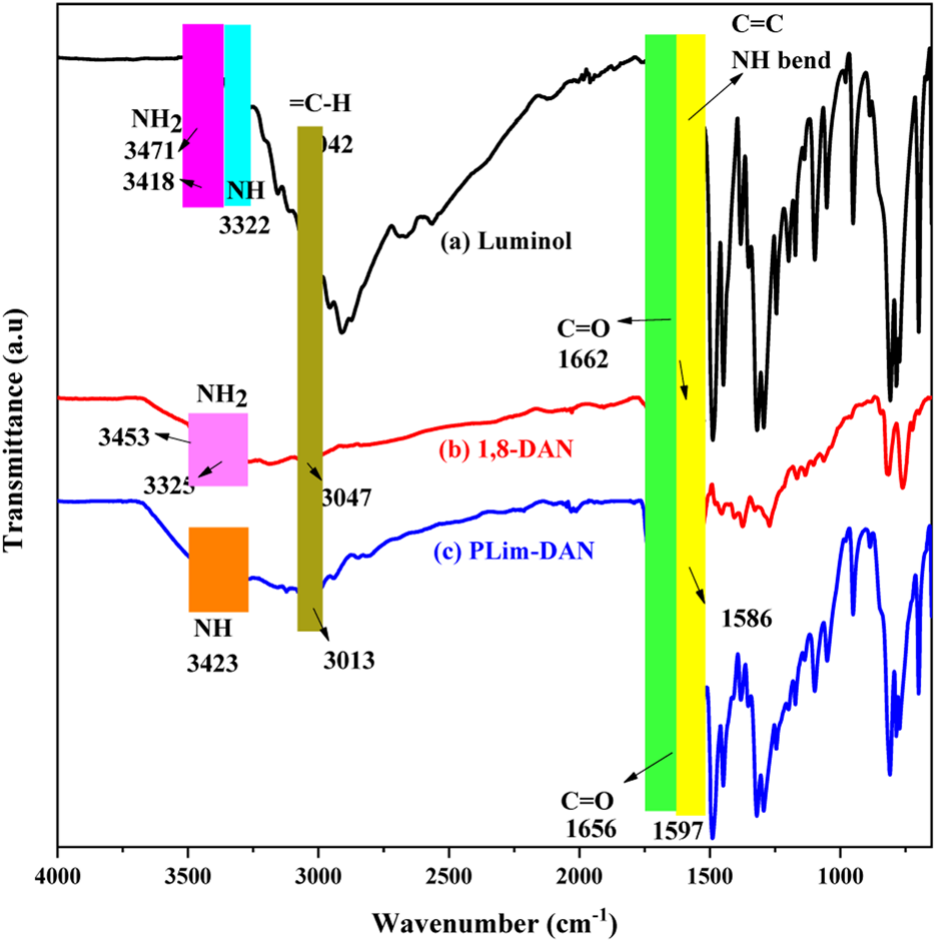
FT-IR spectra for (a) luminol, (b) 1,8-diaminonaphthalene, and (c) copolymer PLim-DAN.

The innovatively synthesized copolymer PLim-DAN and its nanocomposites were investigated using FT-IR analysis. Fig. 2 shows the FT-IR spectra of the two monomers, luminol and 1,8-diaminonaphthalene, compared with the newly designed copolymer PLim-DAN. In the luminol spectra (Fig. 2a), NH_2_ appeared as two bands at 3471 and 3418 cm^−1^, while the NH of the amide group was observed at 3322 cm^−1^. The spectrum of 1,8-diaminonaphthalene (Fig. 2b) shows bands at 3453 and 3325 cm^−1^ associated with the amino group. For the pure copolymer (Fig. 2c), the spectrum demonstrates the absence of NH_2_ bands overlapping with the NH peak, confirming the formation of a polymeric bond.^12,52^ The C–H symmetric aromatic stretching band appeared in monomers and the copolymer in the 3004–3083 cm^−1^ range. The amide group C=O band was present in the luminol spectrum (Fig. 2a) at 1662 cm^−1^ and in the copolymer at 1656 cm^−1^ (Fig. 2c). The bands at 1593 and 1586 cm^−1^ in monomers and at 1597 cm^−1^ in the copolymer represent symmetric and asymmetric aromatic ring stretching of(C=C) overlapped with the vibrations bending of (N–H) amide group, while the peaks located between 809–818 cm^−1^ in (Fig. 2a–c) may be related to aromatic C–H bending. The fingerprint area bands of the copolymer spectrum combined both bands in luminol and 1,8-diaminonaphthalene with a broad shape.^54,55^

**Fig. 3:**
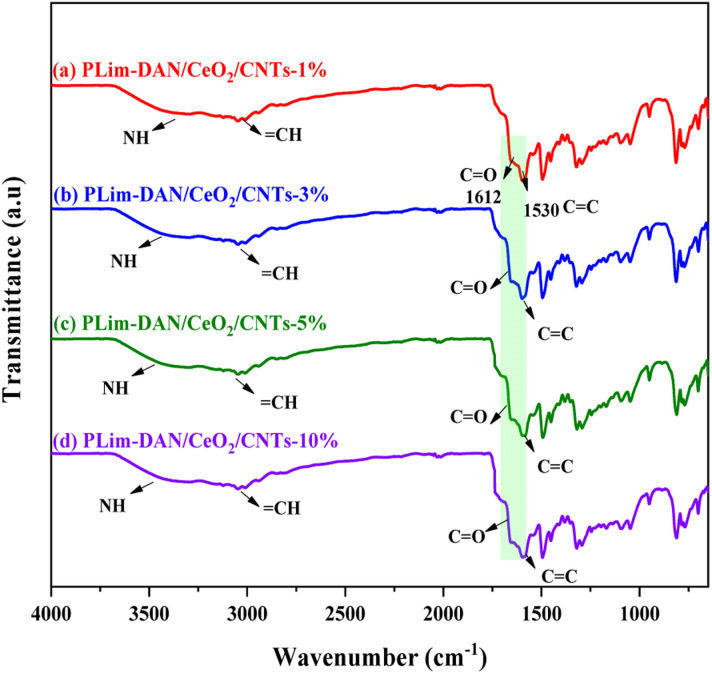
FT-IR spectra for (a) PLim-DAN/CeO_2_/CNTs-1%, (b) PLim-DAN/CeO_2_/CNTs-3%, (c) PLim-DAN/CeO_2_/CNTs-5% and (d) PLim-DAN/CeO_2_/CNTs-10% nanocomposites.

Fig. 3 presents the FT-IR spectra of the nanocomposites PLim-DAN/CeO_2_/CNTs-1%, PLim-DAN/CeO_2_/CNTs-3%, PLim-DAN/CeO_2_/CNTs-5%, and PLim-DAN/CeO_2_/CNTs-10%. In all spectra (Fig. 3a–d), the band at 1612 cm^−1^ was assigned to the C=C stretching vibration of the MWCNTs. In contrast, the broad peak at 1530 cm^−1^ was attributed to the C–C plane vibrations of the graphitic walls of the MWCNTs.^53^ The peak at 500 cm^−1^ might be correlated with the Ce–O stretching vibration. The major bands of the copolymer (Fig. 2c) and those related to the MWCNTs appeared in the spectra of the other nanocomposites (Fig. 3a–d). The FT-IR characterization confirmed the successful formation of the copolymer and its nanocomposites. Nevertheless, compared to the unfabricated copolymer, the nanocomposite spectra show similar peaks regions only with deformation in presentation due to the coating effect of MWCNTs and CeO_2_ NPs, impacting the vibrational mode.^56^

### Morphology analysis

**Fig. 4:**
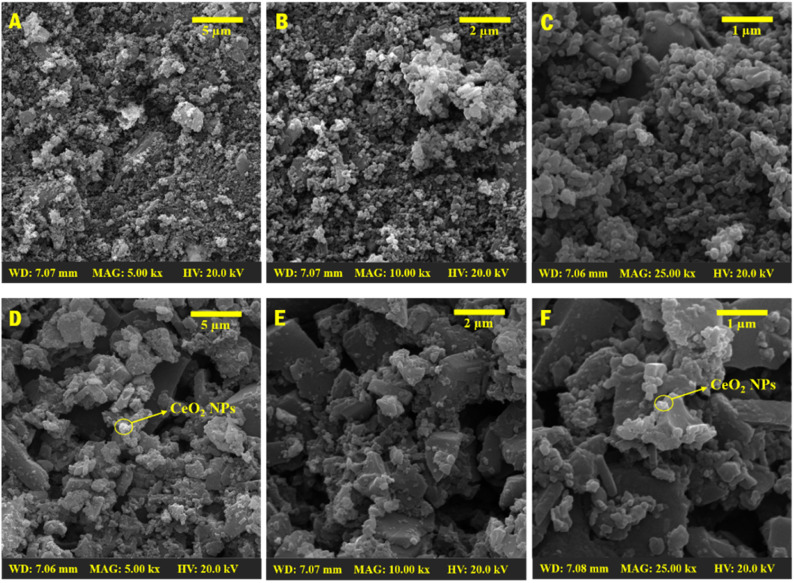
SEM images of pure copolymer PLim-DAN (A–C) and PLim-DAN/CeO_2_/CNTs-10% nanocomposites (D–F) (× = 5000, 10 000, and 25 000).

Systematic analysis using SEM was conducted at different magnifications between 5k× to 25k× and scale bar from 5 μm to 1 μm, to elucidate further the surface morphologies of the newly synthesized copolymer and its nanocomposites. Fig. 4 presents the SEM images of the bare copolymer PLim-DAN (Fig. 4a–c) and PLim-DAN/CeO_2_/CNTs-10% nanocomposite (Fig. 4d–f). The pure copolymer matrix (Fig. 4a–c) displays an irregular spherical morphology encompassing large and small particles with no discernible dots or spots on the surface. Some spherical grains aggregated in certain areas, which became clearer at high magnification (Fig. 4c). On the other hand, Fig. 4d–f of PLim-DAN/CeO_2_/CNTs-10% displays scattered bright spots as small aggregated globules appearing on the surface, signifying the existence of cerium oxide nanoparticles (CeO_2_ NPs).^57,58^ Furthermore, the images revealed the dispersion and coating of MWCNT nanofillers, with the polymer surface irregularly shaped growth on the MWCNTs being much denser and thicker for PLim-DAN/CeO_2_/CNTs-10% with clustered MWCNTs on the surface.^59,60^ The nanofiller (MWCNT) had a large surface-to-volume ratio, which provided sorption sites for the luminol and 1,8-diaminonaphthalene monomers that polymerized, resulting in significant coverage of the MWCNTs.^61^

**Fig. 5:**
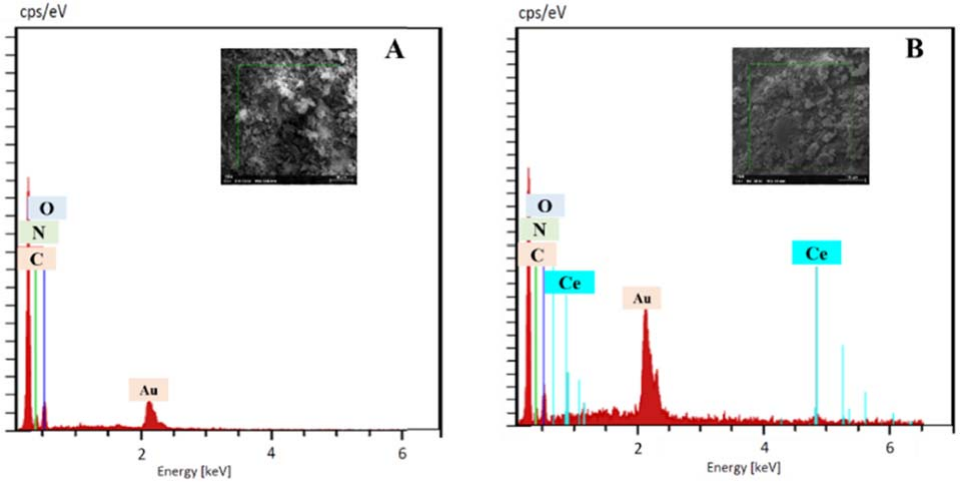
EDX spectroscopy of pure copolymer PLim-DAN (A) and PLim-DAN/CeO_2_/CNTs-10% nanocomposites (B).

The elemental compositions of the synthesized copolymer PLim-DAN and the PLim-DAN/CeO_2_/CNTs-10% nanocomposites were analyzed using electron diffraction X-ray analysis (EDX). The EDX spectrum showed the presence of C, N, and O in the unmodified copolymers (Fig. 5a).

**Fig. 6:**
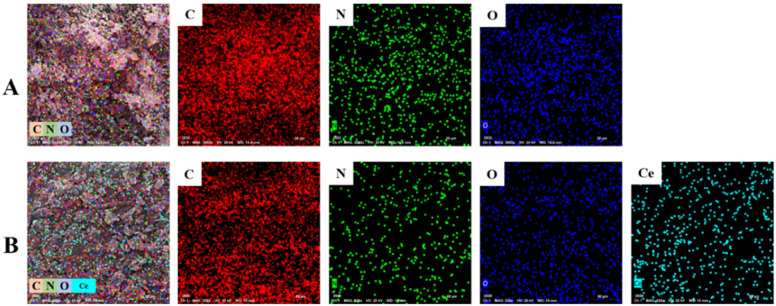
EDX elemental maps of (A) pure copolymer PLim-DAN (C, N and O), and (B) PLim-DAN/CeO_2_/CNTs-10% nanocomposites (C, N, O and Ce).

Meanwhile, PLim-DAN/CeO_2_/CNTs-10% nanocomposite confirmed the loading of CeO_2_ NPs by demonstrating an additional peak attributed to Ce (Fig. 5b). Notably, the presence of the Au peak in the EDX spectra can be attributed to the gold coating applied to the samples before analysis. Moreover, Fig. 6a and b illustrate the elemental mapping of the pure copolymer PLim-DAN and PLim-DAN/CeO_2_/CNTs-10%, respectively. As seen in Fig. 6a, the C, O, and N elements were homogeneously distributed on the copolymer matrix. In contrast, the mapping in Fig. 6b exhibits an additional Ce element uniformly dispersed on the surface of the nanocomposite. The SEM/EDX outcomes established the successful synthesis of the copolymer and its nanocomposite through *in situ* oxidative polymerization.

**Fig. 7:**
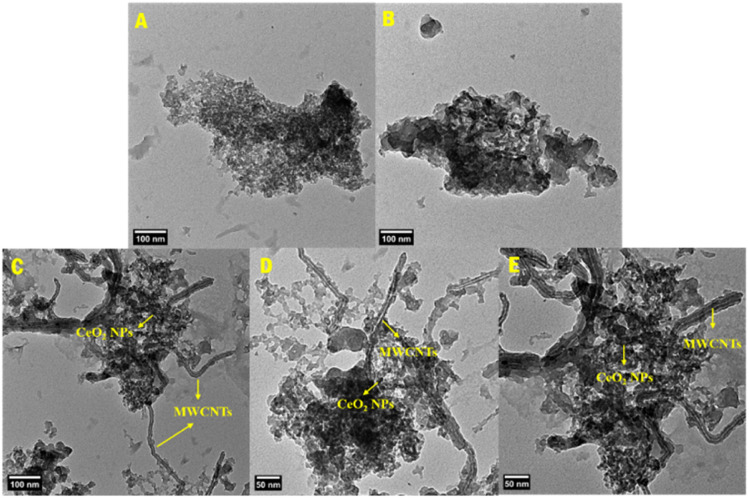
TEM images of pure copolymer PLim-DAN (A and B) and PLim-DAN/CeO_2_/CNTs-10% nanocomposites (C–E).

The TEM technique was studied to provide a more detailed description of CeO_2_ NPs and MWCNT dispersion within the synthesized copolymer matrix in the form of nanocomposites. TEM images of the PLim-DAN and PLim-DAN/CeO_2_/CNTs-10% nanocomposites are presented in Fig. 7. The pure copolymer displayed conglomeration with an irregular spherical morphology (Fig. 7a and b).

The copolymer network in the MWCNT host showed strong interactions in the form of thin, dark threads that spread in the nanocomposite backbone of PLim-DAN/CeO_2_/CNTs-10% (Fig. 7c–e), confirming the intercalation of MWCNTs into the PLim-DAN matrix and the small dark spots assigned to the presence of CeO_2_ NPs.^58–60^ The stable intersection of the nanofiller enhanced the mass transport and electron transfer between the MWCNT and PLim-DAN copolymers through donor–acceptor interplay, which improved the electrochemical performance of the prepared nanocomposites.^62,63^

**Fig. 8:**
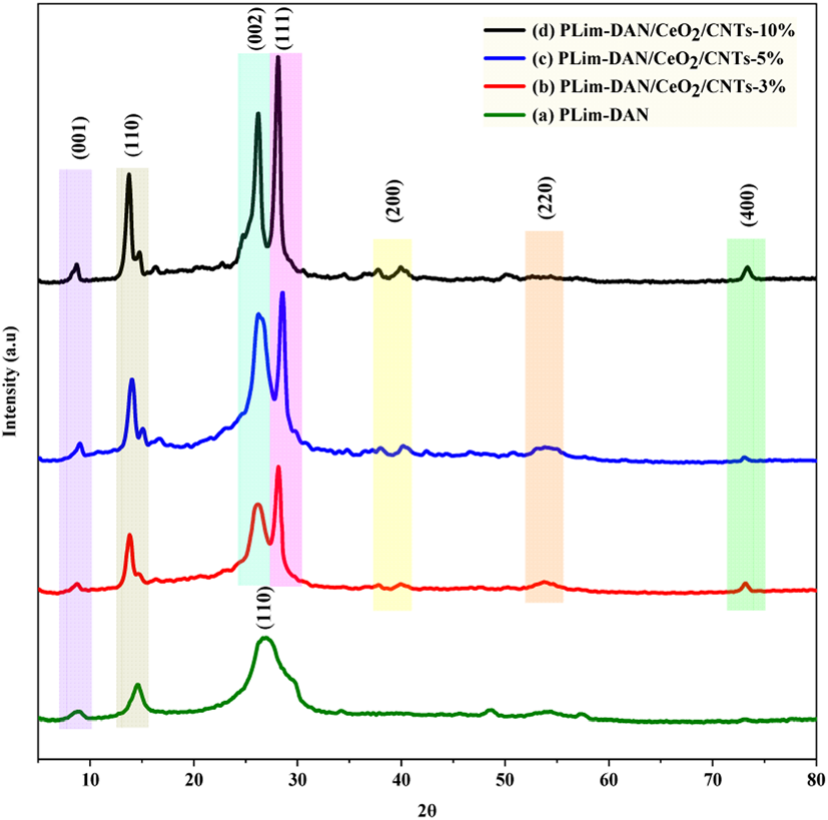
XRD diffraction patterns for (a) PLim-DAN, (b) PLim-DAN/CeO_2_/CNTs-3%, (c) PLim-DAN/CeO_2_/CNTs-5%, and (d) PLim-DAN/CeO_2_/CNTs-10% nanocomposites.

The proposed structures of the pure copolymer and its nanocomposites were inspected using XRD, as illustrated in Fig. 8, to understand the crystalline nature and loading of nanoparticles on the polymeric matrix. The crystallographic patterns of the pure copolymer and nanocomposites exhibited diffraction lines of a primarily crystalline nature. All spectra showed peaks at the low angle region between 6° and 25° which may indicate to the formation of ordered lamellar in the crystalline phase for polymeric (luminol-*co*-1,8-diaminonaphthalene) and the nanocomposites as (001) diffraction peaks at 2*θ* = 8.6° and the (110) plane at 2*θ* = 14.5° and 26° as main peaks for the π–π stacking distance of polymer network.^64,65^ Furthermore, the literature indicates that the primary diffraction peaks at between 2*θ* = 14° and 25° are associated with periodicities parallel and perpendicular to the polymer chains.^66^ The XRD pattern of the pure copolymer PLim-DAN (Fig. 8a) revealed that the copolymer featured a semi-amorphous form with broad peaks of (001), (110) at 8°, 14° and 26°.^54,55^ The spectra of PLim-DAN/CeO_2_/CNTs-3%, PLim-DAN/CeO_2_/CNTs-5%, and PLim-DAN/CeO_2_/CNTs-10% nanocomposites (Fig. 8b–d) show the same bands in the low angle region and extra bands at 28°, 37°, 54°, 73° are ascribed to the (111), (200), (220), and (400) lattice planes of the cubic structure of CeO_2_ nanoparticles, demonstrating successful loading of NPs and in accordance with the data registered in the literature (JCPDS 43-1002).^67,68^ Nevertheless, the studied nanocomposites (Fig. 8b–d) display the diffraction pattern of MWCNTs appearing at 2*θ* values of 26° (002), corresponding to reflections of the pseudo-graphite structure.^69^ The peak intensity increased gradually with increasing MWCNT loading from 1% to 10%, confirming the insertion of MWCNTs into the copolymer molecules and the successful dispersion of the filler into the polymer matrix. Above that, the diffraction spectra revealed the effect of MWCNTs on the crystallinity of the nanocomposites. The characteristic diffraction peaks became sharper than before with the increase in the MWCNT loading, clearly confirming the crystalline phase of the nanocomposite.^70^ The results were consistent with the outcomes obtained from the thermal analyses, where thermal stability increased with increasing MWCNT loading.

### Thermal analysis

**Fig. 9:**
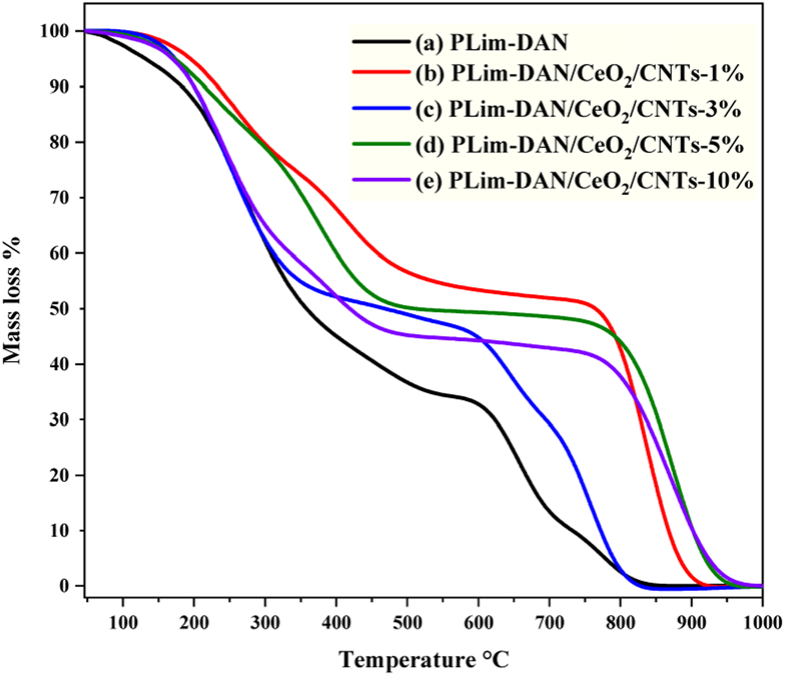
TGA curves of (a) PLim-DAN (b–e) PLim-DAN/CeO_2_/CNTs nanocomposites.

**Fig. 10:**
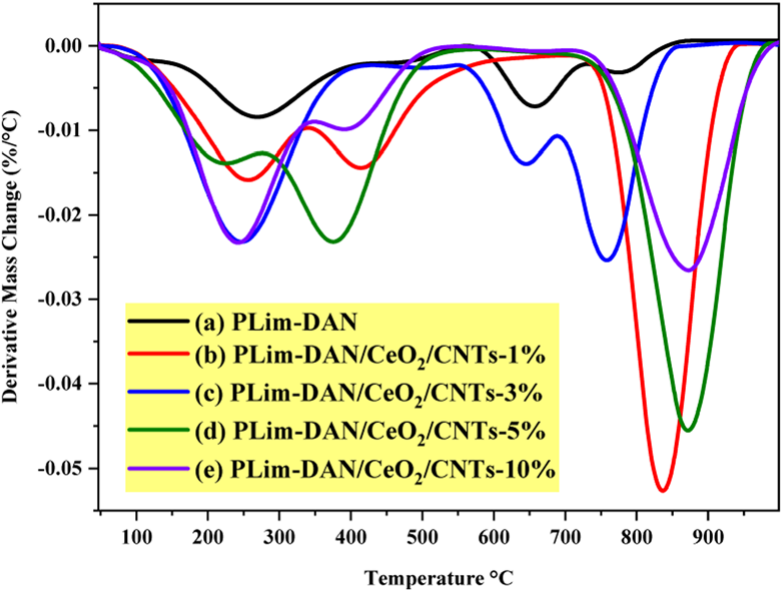
DTG curves of (a) PLim-DAN (b) PLim-DAN/CeO_2_/CNTs nanocomposites (b–e).

The thermal performance of the synthesized copolymers and nanocomposites was studied using TGA and derivative thermogravimetry (DTG) in the temperature range of 25–1000 °C at a heating rate of 10 °C min^−1^ to provide more insights into the possible applications (Fig. 9a–e and 10a–e). The TGA/DTG curve exhibited a three-step weight loss system. The first stage occurred between 25 °C and 280 °C, attributed to moisture withdrawal. The second stage of weight loss followed between 280 °C and 760 °C owing to the degradation of the polymer backbone.^71,72^

The third stage, observed between 760 °C and 940 °C, where the nanocomposites exhibited high thermal stability up to 800 °C for the 10%, 5% and 1% loading of MWCNTs with approximately 50% of the mass loss. Moreover, the nanocomposites (Fig. 9b–e) showed similar thermal performance when a fixed loading of CeO_2_ NPs and different loadings of MWCNTs were introduced to the copolymer, implying that the nanocomposites displayed similar decomposition paths. The nanofiller addition of CeO_2_ NPs and MWCNTs resulted in a shift in the second and third stages of nanocomposite decomposition to higher temperature values. This result suggests that the nanofiller improved the thermal stability of the copolymer.^57,73–75^Table 1 presents a comprehensive comparison of *T*_10_, *T*_25_, and *T*_50_, illustrating thermal decomposition at 10%, 25%, and 50%, respectively. The *T*_10_, *T*_25_, and *T*_50_ values indicated a pattern performance controlled by the amount of MWCNTs loaded. As illustrated in Fig. 9 and Table 1, the increase in the percentage of the nanofiller was matched by an increase in thermal decomposition at 10%, 25%, and 50%.

**Table 1:** Thermal behavior of PLim-DAN and PLim-DAN/CeO_2_/CNTs nanocomposites

Sample	Temperature °C for various percentage decompositions^a^			PDT_final_^a^ °C	PDT_max_^b^ °C
	*T*_10_	*T*_25_	*T*_50_		
PLim-DAN	180	248	357	815	272
PLim-DAN/CeO_2_/CNTs-1%	233	341	764	921	837
PLim-DAN/CeO_2_/CNTs-3%	201	252	464	828	757
PLim-DAN/CeO_2_/CNTs-5%	213	327	703	940	870
PLim-DAN/CeO_2_/CNTs-10%	201	257	427	950	874

a The values were determined by TGA at a heating rate of 10 °C min^−1^.

b The values were determined from the DTG curves.

Table 1 outlines the final polymer degradation temperature (PDT_final_) and the maximum polymer decomposition temperature (PDT_max_). As exemplified in Table 1, the PLim-DAN/CeO_2_/CNTs-1% nanocomposite showed high optimum stability at *T*_10_, *T*_25_, and up to *T*_50_ from a thermal point of view. As the table and TGA clarified, the PDT_final_ values ranged from 815 °C to 950 °C, while the DTG revealed PDT_max_ in the 272–874 °C. The nanocomposite PLim-DAN/CeO_2_/CNTs-3% demonstrated the lowest values of PDT_final_ and PDT_max_ in comparison with the other nanocomposites, whereas PLim-DAN/CeO_2_/CNTs-10% displayed the highest values at both degradation temperatures.

### Electrochemical study

**Fig. 11:**
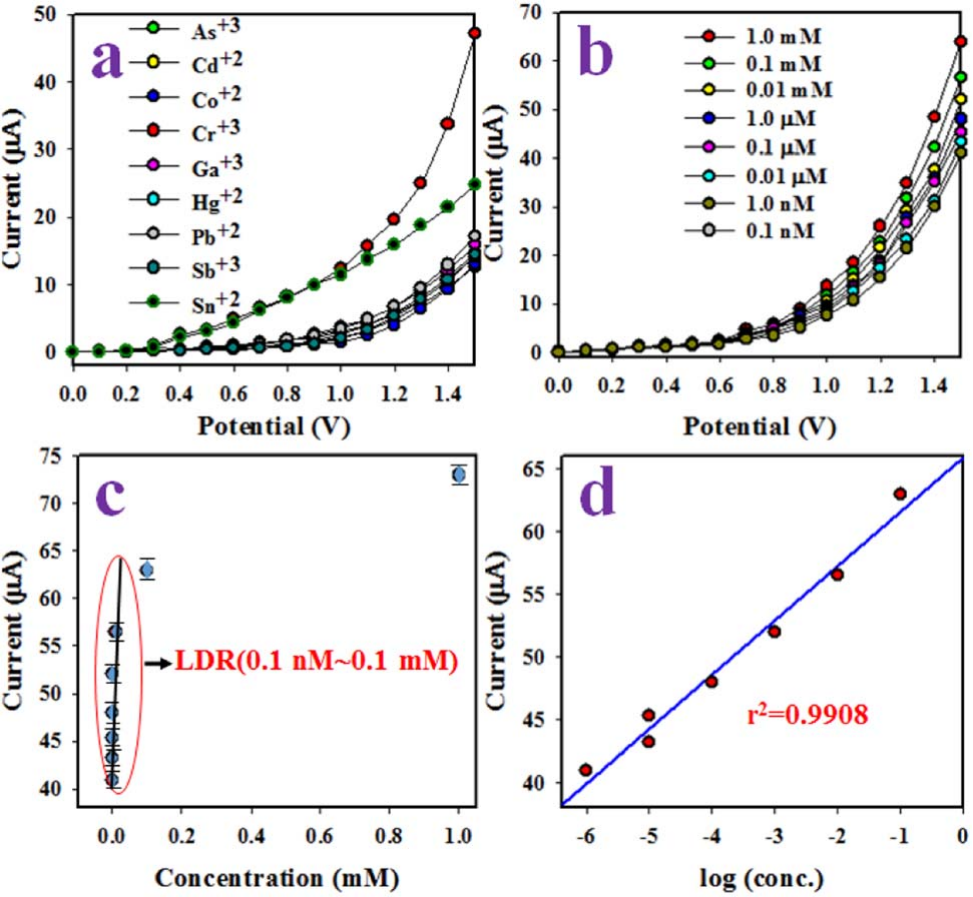
Identification of sensor behavior using the electrochemical (I–V) approach. (a) Estimation of selectivity, (b) I–V responses based on the variation in the concentration of Cr^3+^ ions from lower to higher, (c) calibration curve, and (d) investigation of the linearity of LRD.

Detection of chromium ions (Cr^3+^) employing PLim-DAN/CeO_2_/CNTs NCs an electrochemical sensor that could specifically detect Cr^3+^ ions was manufactured using a glassy carbon electrode (GCE) and a composite of active PLim-DAN/CeO_2_/CNTs-10% NCs. A Nafion conducting binding agent was employed to create a thin, uniform layer on the GCE. Nafion improves the adhesion of the PLim-DAN/CeO_2_/CNTs NCs to the GCE and enhances the electron transfer rate of the desired electrochemical sensor through I–V analysis.^76,77^ The resulting electrochemical sensor demonstrated good sensitivity, a meager DL, a wide LDR, and long-term stability in a phosphate buffer medium with good reproducibility. During the initial stages of the I–V study, several heavy metal ions at a concentration of 0.1 μM and an applied potential of 0 to +1.5 V were analyzed in a phosphate buffer medium with a pH of 7.0. Cr^3+^ displays the highest I–V response among the electrochemical responses of As^3+^, Cd^2+^, Co^2+^, Cr^3+^, Ga^3+^, Hg^2+^, Pb^2+^, Sb^3+^, and Sn^2+^ ions (Fig. 11a).

The Cr^3+^ ion solution with concentrations ranging from 1.0 mM to 0.1 nM was investigated (Fig. 11b). The electrochemical responses were visibly distinguishable at different concentrations of Cr^3+^ ions from lower to higher. The analytical performance of the projected Cr^3+^ ions based on the PLim-DAN/CeO_2_/CNTs-10% NCs/GCE was determined by plotting a calibration curve between the current and the concentration of Cr^3+^ ions, as shown in Fig. 11c. The measured slope of the calibration curve was used to determine the sensitivity and DL of the Cr^3+^ sensor. The obtained values were 19.78 μA μM^−1^ cm^−2^ and 4.80 ± 0.24 pM, respectively.

Furthermore, a contentious distribution of the current data at an applied potential of +1.5 V along the linear plot over the concentration range of 0.1 nM to 0.1 mM was obtained (Fig. 11c), signifying the LDR. LDR was determined to have a significantly wide range of concentrations. A curve of the current *vs.* log(concentration of Cr^3+^ ions) was plotted to assess the linearity of LDR (Fig. 11d). The current data were fitted with a regression coefficient of *R*^2^ = 0.9908, providing evidence of linear LDR.

**Fig. 12:**
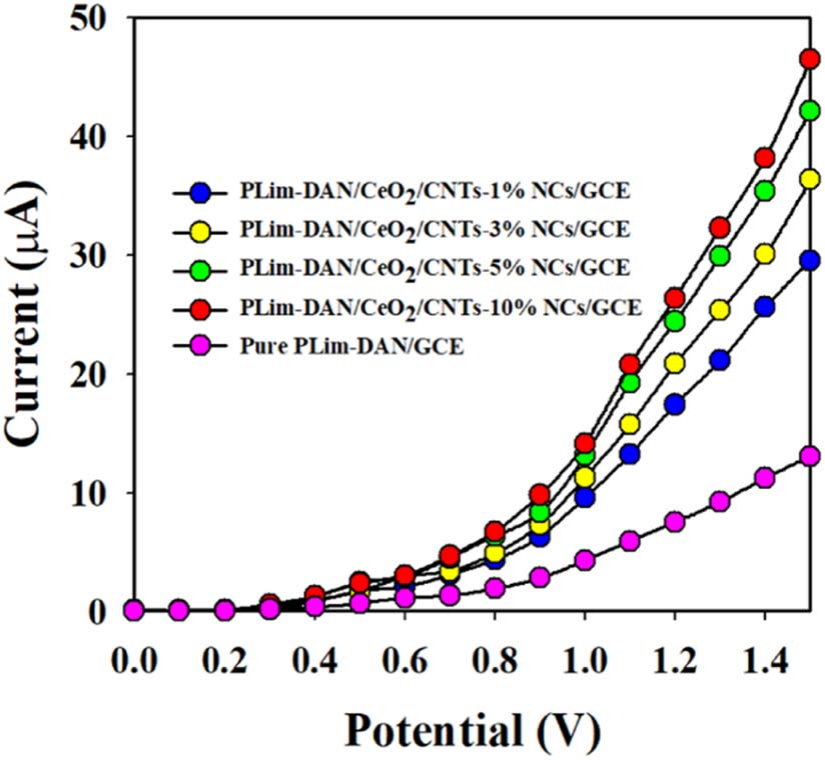
A control experiment executed at 0.1 μM Cr^3+^ solutions in a buffer medium with modified GCE containing PLim-DAN and a 1–10% MWCNT composition of nanocomposites.

The control experiment (Fig. 12) was conducted using 0.1 μM Cr^3+^ solutions in a buffer environment with modified GCE containing the pure copolymer and diverse percentages of MWCNT compositions (1–10%). The adapted GCE with the PLim-DAN/CeO_2_/CNTs-10% NCs exhibited the highest current response among the other compositions and pure PLim-DAN. Hence, a 10% composition of CNT is the optimum composition for studying Cr^3+^ ions using an electrochemical method.

**Fig. 13:**
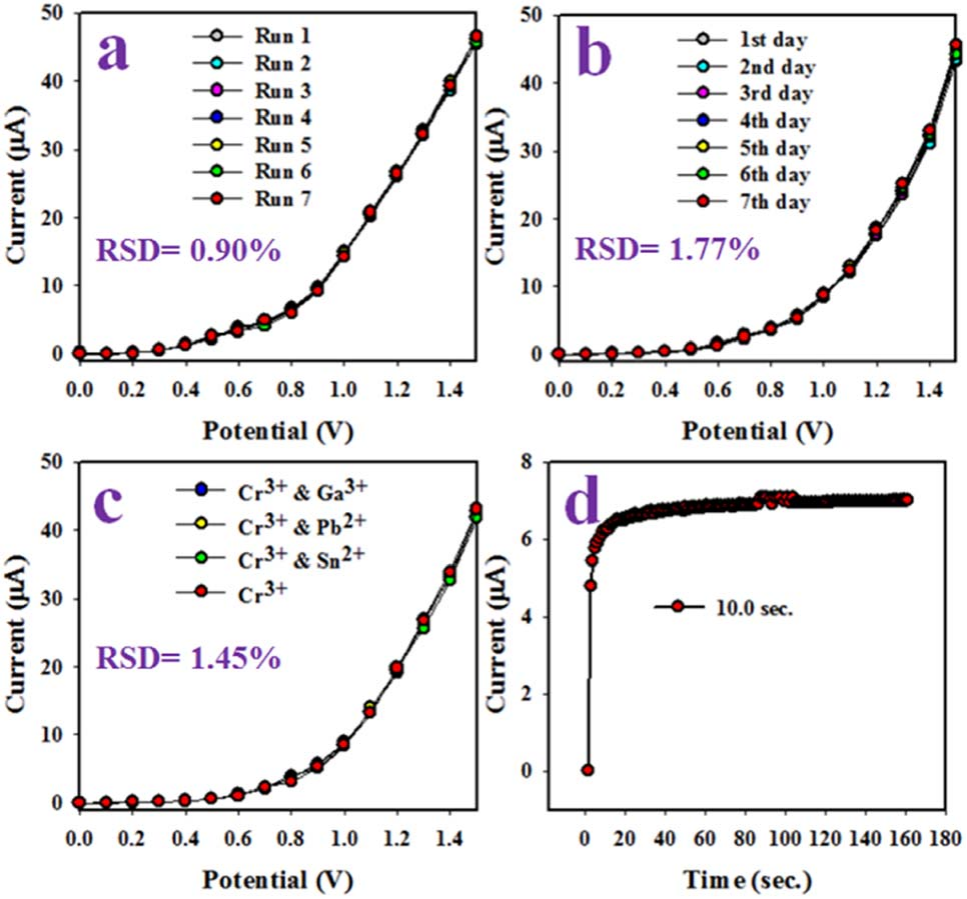
(a) reproducibility test, (b) validity test of the Cr^3+^ ion sensor based on PLim-DAN/CeO_2/_CNTs-10% NCs/GCE, (c) interference effect estimation, and (d) response time.

The reproducibility of the electrochemical sensor is a crucial reliability test. The test was performed using a 0.1 μM concentration of Cr^3+^ ion solution and an applied potential ranging from 0 to +1.5 V (Fig. 13a). The results displayed a perfectly indistinguishable (I–V) response, and the seven replicated runs showed no signs of alteration, even after washing the electrode following each run. These results confirm the reliability of the proposed Cr^3+^ ion sensor. The relative standard deviation (RSD) of the current data at an applied potential of +1.5 V was measured to evaluate the accuracy of this reproducibility test. As a result, a significant RSD value of 0.90 was obtained.

Similarly, a reproducibility test was conducted over approximately seven days to evaluate the stability and performance of the Cr^3+^ ion sensor.

The Cr^3+^ ion sensor displayed consistent results over a long period, with a % RSD of 1.77 (Fig. 13b). This result confirms the long-term stability of the Cr^3+^ ion sensor to a constant outcome of the I–V response. An interference test of the Cr^3+^ ion sensor based on PLim-DAN/CeO_2_/CNTs-10% NCs/GCE was conducted (Fig. 13c). The findings exhibited high selectivity for Cr^3+^ ion sensors with no interference effects caused by other cations, such as Ga^3+^, Pb^2+^, and Sn^2+^. Additionally, the response time of the sensor was measured to be approximately 10.0 seconds when a 0.1 μM concentration of Cr^3+^ ions was used in a phosphate buffer medium, as depicted in Fig. 13d. Overall, the sensor demonstrated adequate efficiency and performance.

The Cr^3+^ ion electrochemical sensor based on PLim-DAN/CeO_2_/CNTs-10% NCs/GCE demonstrated impressive performance with high sensitivity (19.7785 μA μM^−1^ cm^−2^), a wide LDR (0.1 nM to 0.1 mM), and a meager DL (4.80 ± 0.24 pM). Furthermore, the designed sensor exhibited reliable and consistent performance with a short response time (10.0 s) and excellent long-term stability in a phosphate buffer medium, making it a good candidate for assessment in actual environmental samples. The response and recovery times of the target PLim-DAN/CeO_2_/CNTs-10% NCs/GCE sensor probe in the detection of target Cr^3+^ analyte by electrochemical method for 10 repetitive test and are included in Table 2.

**Table 2:** Summarized sensing results of target PLim-DAN/CeO_2_/CNTs-10% based fabricated sensor-probe towards different concentration of target analyte (Cr^3+^) ions by electrochemical technique in terms of sensing response, sensing time, recovery time, and reversibility

Trials	Analyte conc.	Sensing response (%)	Sensing time (s)	Recovery time (s)	Reversibility (%)
1	10.0 mM	98.3	14	17	96.3
2	1.0 mM	97.2	15	16	97.2
3	0.10 mM	96.1	13	15	91.6
4	10.0 μM	97.4	14	16	95.4
5	1.0 μM	95.6	12	11	94.7
6	0.1 μM	97.8	10	12	95.4
7	0.01 μM	93.2	13	14	91.8
8	1.0 nM	87.5	15	18	88.7
9	0.1 nM	84.0	14	15	79.2
10	0.01 nM	83.9	13	14	81.2

Table 3 shows a comparison of the results obtained from the PLim-DAN/CeO_2_/CNTs NCs/GCE modified sensor and several recently reported investigations in the literature for the detection of Cr^3+^ ions. The table comprises the modified material on the electrode, linear dynamic range, detection limits, and real sample test. As the table exemplified, the electrochemical sensor, purposely designed for the detection of Cr^3+^ ions in this study, exhibits a low detection limit and a wide linear range. Additionally, the fabricated sensor reported here registered a lower detection limit than our previous research using the same (I–V) method.^83^

**Table 3:** Comparison of the performances of different electrochemical sensors for the determination of Cr^3+^^a^

Modification material/electrode	Technique	Linear dynamic range	Limit of detection	Real sample composition	Ref.
Chitosan-Au/SPCE	DPSV	1–100 μM	0.4 μM	Industrial wastewater	78
IIP (itaconic acid based)/CPE	DPV	0.1–10 μM	17.6 nM	River/sea water, urine	79
AgNP-LE/Pt	DPV	10–90 μM	0.804 μM	Lake/waste water	80
AuNPs/SPCE	LSV	0.5–10 μM	0.01 μM	Tea, coffee and mineral water	81
Bi film/GCE	FSV	10^−12^ to 10^−7^ M	0.3 pM	Tap water	82
4-BBBSH/GCE	I–V	100 pM to 100 mM	95.5 pM	Coal/Red Sea/tap/well water, industrial effluent	83
AuNPs-mercapto succinic acid/ITO	ASV	0.1–10 nM	0.05 nM	Tap/pond/lake water	84
AuNP-3-mercaptopropionic acid/ITO	SWV	200–500 ppb	278 ppb	Pond water	85
PLim-DANCeO_2_/CNTs/GCE	I–V	0.1 nM to 0.1 mM	4.80 pM	Industrial effluent/sea water/ground mineral water	This work

a SPCE: screen-printed carbon electrode; DPSV: differential pulse stripping voltammetry; CPE: carbon paste electrode; DPV: differential pulse voltammetry; LSV: linear sweep voltammetry; FSV: fast scan voltammetry; 4-BBBSH: (*E*)-*N*′-(4-bromobenzyledene)-4-benzenesulfonohydrazine; ITO: indium-doped tin oxide; ASV: anodic stripping voltammetry; SWV: square wave voltammetry.

### Potential mechanism for the detection of Cr(iii)

**Fig. 14:**
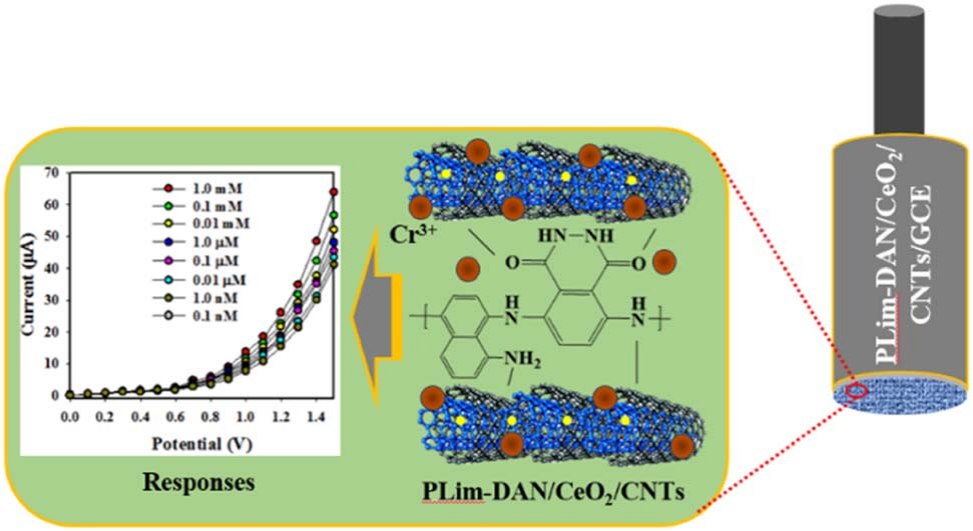
Fabrication and mechanism of the probable interaction of Cr^3+^ with PLim-DAN/CeO_2_/CNTs-10%/GCE with conducting 0.5% Nafion binders embedded onto GCE.

In this approach, the sensing mechanism can be explained as follows: the electrochemical detection of the PLim-DAN/CeO_2_/CNTs is worked as a function of Cr^3+^ ions concentration interaction onto the fabricated surface at room conditions. The improved current response is observed with the fabricated electrode and probable mechanism is included in the Fig. 14. As obtained, the current response of the PLim-DAN/CeO_2_/CNTs-10% film is significantly increased with the increasing concentration of target Cr^3+^ ions due to the large surface area of PLim-DAN/CeO_2_/CNTs-10%, as well as electrochemical interaction and adsorption the target cations onto the sensor functional surface of PLim-DAN. The similar phenomena for toxic chromium ions detection with various nanocomposite materials have also been reported elsewhere.^86,87^ For a low concentration of Cr^3+^ ions in liquid medium, there is a smaller surface coverage of Cr^3+^ ions on PLim-DAN/CeO_2_/CNTs-10%/GCE film and hence the surface reaction proceeds steadily. By increasing the Cr^3+^ ions concentration, the surface reaction is increased significantly (gradually increased the response) owing to surface area (assembly of PLim-DAN/CeO_2_/CNTs-10%) contacted with Cr^3+^ ions (Fig. 14). Further increase of Cr^3+^ ions concentration onto PLim-DAN/CeO_2_/CNTs-10%/GCE surface, it is exhibited a more rapid increasing the current responses, due to larger area covered by Cr^3+^ ions as well as the electrochemical interaction of the nitrogen and oxygen containing functional groups (Fig. 14) with the target cations. The interaction could be approaches as inter-molecular and intra-molecular interactions of the PLim-DAN layer with the target analyte.^88^ Usually, the surface coverage of Cr^3+^ ions onto PLim-DAN/CeO_2_/CNTs-10%/Naf/GCE surface is reached to the saturation level, based on the regular enhancement of current responses.

### Real environmental samples analysis

The PLim-DAN/CeO_2_/CNTs-10% NCs/GCE for the Cr^3+^ ion electrochemical sensor was utilized to examine various real environmental samples, including industrial waste effluent, ground mineral water, and seawater. Considerable results were obtained, as summarized in Table 4.

**Table 4:** Analyses of environmental samples with the PLim-DAN/CeO_2_/CNTs-10% NCs/GCE sensor

Samples	Added Cr^3+^ ion concentration (μM)	Determined Cr^3+^ conc.^a^ by PLim-DAN/CeO_2_/CNTs-10% NCs/GCE (μM)			Average recovery^b^ (%)	RSD^c^ (%) (*n* = 3)
		*R*1	*R*2	*R*3		
Industrial effluent	0.01000	0.009339	0.009689	0.0091616	93.97	2.86
Sea water	0.01000	0.009834	0.009627	0.009844	97.68	1.25
Ground mineral water	0.01000	0.009649	0.009908	0.009900	98.19	1.50

a Mean of three repeated determinations (signal-to-noise ratio of 3) with PLim-DAN/CeO_2_/CNTs-10% NCs/GCE.

b Concentration of Cr^3+^ ions determined/concentration taken (unit: μM).

c Relative standard deviation value indicates precision among three repeated measurements (*R*1, *R*2, and *R*3).

**Fig. 15:**
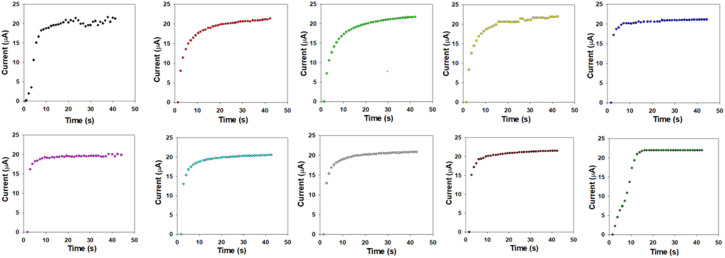
Sensor responses towards target analyte (Cr^3+^) ions with prepared PLim-DAN/CeO_2_/CNTs nanocomposite in identical conditions.

**Fig. 16:**
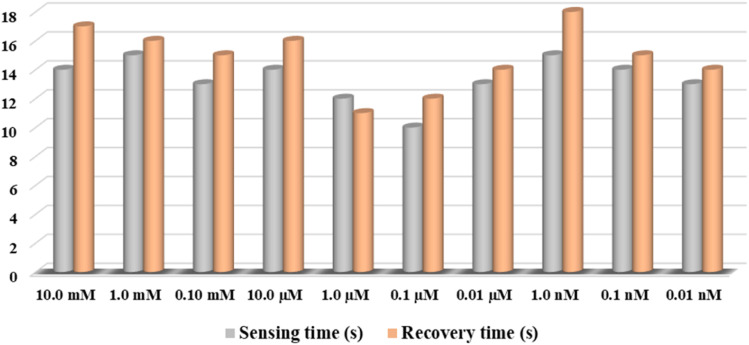
The response time *vs.* recovery time for the modified PLim-DAN/CeO_2_/CNTs-10% sensor during the range of10.0 mM to 0.01 nM concentration.

Furthermore, the PLim-DAN/CeO_2_/CNTs-10% based fabricated sensor-probe response time *vs.* current has been calculated and illustrated in Fig. 15 for each concentration.^89,90^Fig. 16 presented a relation between response time and recovery time at different concentration between 10.0 mM to 0.01 nM. As shown in Table 2 and Fig. 16, the 0.1 μM of Cr^3+^ solution displayed the fastest response time of 10 s and 12 s of recovery time with 97.8% of the sensing response.

## Conclusion

Copolymerization of luminol and 1,8-diaminonaphthalene was designed and quaternary PLim-DAN/CeO_2_/CNTs nanocomposites with different loading values (1, 3, 5, and 10%) were successfully modified and characterized in this work. XRD diffraction spectra and TGA revealed the effect of MWCNTs on the crystallinity and thermal stability of the nanocomposites, whereby increasing the MWCNT loading sharpens the characteristic diffraction peaks. This result confirms the crystalline phase of the nanocomposite, along with its high thermal stability. TEM images revealed strong interactions between PLim-DAN and MWCNTs, enhancing the efficiency of mass transport and electron transfer in electrochemical sensing applications. The electroactivity of the designed nanocomposites toward different heavy metal ions was studied, and the results showed a high sensitivity to Cr^3+^ ions. The GCE modified with the PLim-DAN/CeO_2_/CNTs-10% NCs exhibited the highest current response among the other compositions. The chromium ion sensor was fabricated by attaching PLim-DAN/CeO_2_/CNTs-10% NCs onto a GCE with a conducting Nafion-binding agent. The sensor was then used to detect Cr^3+^ ions in a phosphate buffer solution, showing excellent analytical performance regarding sensitivity, linear dynamic range, and DL. The sensor was also reliable, with a short response time and reproducible results.

## Author contributions

S. Al-Sodies: methodology, investigation, writing – original draft & editing; M. M. Alam: methodology; K. A. Alamry: conceptualization, investigation; M. A. Hussein: conceptualization, investigation, writing – review & editing; A. M. Asiri: review & editing – final draft; M. M. Rahman: methodology, investigation, writing – review & editing.

## Conflicts of interest

There are no conflicts to declare.
